# Clinicopathologic Implications of Complement Genetic Variants in Kidney Transplantation

**DOI:** 10.3389/fmed.2021.775280

**Published:** 2021-11-29

**Authors:** Zhen Ren, Stephen J. Perkins, Latisha Love-Gregory, John P. Atkinson, Anuja Java

**Affiliations:** ^1^Division of Allergy and Immunology, Department of Medicine, Washington University School of Medicine, St. Louis, MO, United States; ^2^Department of Structural and Molecular Biology, Division of Biosciences, University College London, London, United Kingdom; ^3^Genomic and Pathology Services, Department of Pathology and Immunology, Washington University School of Medicine, St. Louis, MO, United States; ^4^Division of Rheumatology, Department of Medicine, Washington University School of Medicine, St. Louis, MO, United States; ^5^Division of Nephrology, Department of Medicine, Washington University School of Medicine, St. Louis, MO, United States

**Keywords:** kidney transplantation, complement, complement regulators, atypical hemolytic uremic syndrome, variants of uncertain significance, thrombotic microangiopathy, C3 glomerulopathy

## Abstract

Genetic testing has uncovered rare variants in complement proteins associated with thrombotic microangiopathy (TMA) and C3 glomerulopathy (C3G). Approximately 50% are classified as variants of uncertain significance (VUS). Clinical risk assessment of patients carrying a VUS remains challenging primarily due to a lack of functional information, especially in the context of multiple confounding factors in the setting of kidney transplantation. Our objective was to evaluate the clinicopathologic significance of genetic variants in TMA and C3G in a kidney transplant cohort. We used whole exome next-generation sequencing to analyze complement genes in 76 patients, comprising 60 patients with a TMA and 16 with C3G. Ten variants in complement factor H (*CFH*) were identified; of these, four were known to be pathogenic, one was likely benign and five were classified as a VUS (I372V, I453L, G918E, T956M, L1207I). Each VUS was subjected to a structural analysis and was recombinantly produced; if expressed, its function was then characterized relative to the wild-type (WT) protein. Our data indicate that I372V, I453L, and G918E were deleterious while T956M and L1207I demonstrated normal functional activity. Four common polymorphisms in *CFH* (E936D, N1050Y, I1059T, Q1143E) were also characterized. We also assessed a family with a pathogenic variant in membrane cofactor protein (*MCP*) in addition to *CFH* with a unique clinical presentation featuring valvular dysfunction. Our analyses helped to determine disease etiology and defined the recurrence risk after kidney transplant, thereby facilitating clinical decision making for our patients. This work further illustrates the limitations of the prediction models and highlights the importance of conducting functional analysis of genetic variants particularly in a complex clinicopathologic scenario such as kidney transplantation.

## Introduction

Two prototypical complement-mediated kidney diseases are atypical hemolytic uremic syndrome (aHUS) and C3 glomerulopathy (C3G) (1). Atypical hemolytic uremic syndrome is a primary complement-mediated thrombotic microangiopathy (TMA) characterized by hemolytic anemia, thrombocytopenia, and acute kidney injury (AKI) (2). C3 glomerulopathy is a group of nephritides in which complement activation leads to a predominant C3 fragment glomerular deposition as seen by immunofluorescence (3). It is subclassified as dense deposit disease or C3 glomerulonephritis (C3GN) based on the type and location of deposits as visualized by electron microscopy.

Recent progress in our understanding of the role of complement dysregulation and efficacy of complement blockade has revolutionized the clinical course and outcome of these diseases (4). Therapeutic advances render kidney transplantation as a viable option for patients with aHUS and C3G who previously were considered non-transplantable.

However, many challenges remain. First, aHUS may be mimicked by other disease processes, currently classified under secondary TMAs, which include infections, pregnancy, autoimmune conditions, and graft rejection (3). Kidney transplantation poses an especially problematic setting since there are multiple potential triggers (transplant surgery, drugs, rejection, and infections) for TMA development. Consequently, it can be “tricky” for transplant clinicians to distinguish aHUS from these secondary TMAs. However, the distinction is critical since complement-mediated TMAs are often non-responsive to conservative measures (removal of the offending drug or treating the underlying rejection/infection) (5). Second, both aHUS and C3G have a high risk of recurrence after kidney transplantation and the outcome can vary depending on the underlying complement abnormality (6, 7). Genetic variants in complement proteins are identified in approximately 60% of patients with aHUS, 10–60% of patients with secondary causes, and 30–40% of patients with C3G (8). However, <50% of the identified variants have a known functional consequence and are classified by the American College of Medical Genetics (ACMG) as variants of uncertain significance (VUS). Thus, the inability to ascertain the functional impact of a VUS poses another major barrier for clinical management after kidney transplantation. Finally, decisions about accepting living related donors for these patients can be complicated since there may be risk of recurrent disease in the recipient and *de novo* disease in the donor if the underlying etiology of disease is unclear.

**Figure 1:**
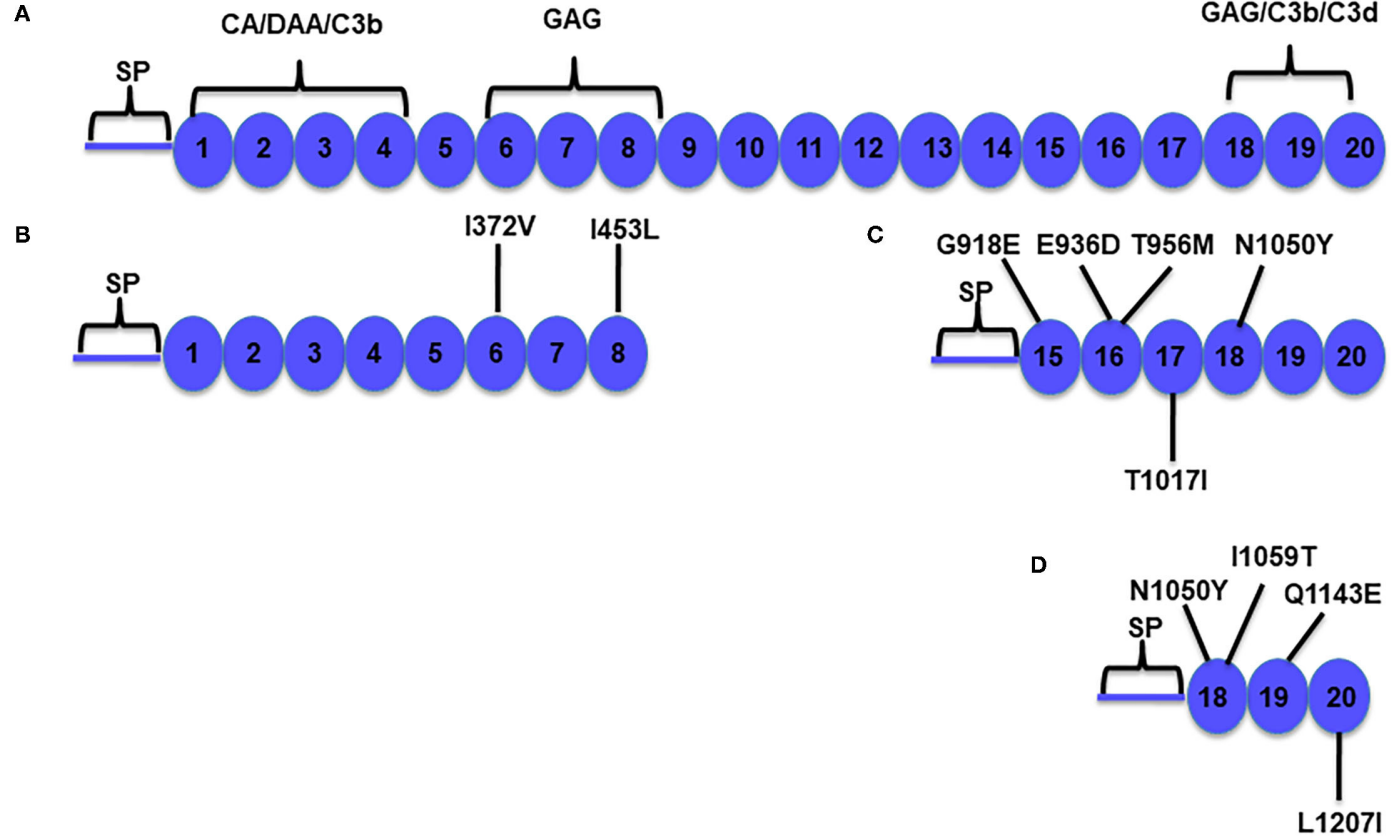
Schematic diagram of Factor H (FH) and its functional domains. **(A)** Factor H is a 155-kDa plasma complement regulatory protein composed of 20 repeating homologous domains of approximately 60 amino acids each, known as complement control protein (CCP) repeats, short consensus repeats (SCRs), or sushi domains (12). Factor H prevents the formation and accelerates the decay of the alternative pathway C3 convertase (C3bBb; decay accelerating activity) and is a cofactor for Factor I-mediated cleavage and inactivation of C3b (cofactor activity). Factor H has fluid-phase and cell-surface regulatory capabilities (13). The N-terminal portion contains the regulatory domain that mediates the cofactor activity and decay accelerating activity (repeats 1–4). The surface binding recognition motifs are located in repeats 6–8 and 18–20. **(B–D)** Factor H constructs used to prepare the variants (as indicated). Residue numbering includes the 18 amino acids of the signal peptide (SP). CA, cofactor activity; DAA, decay accelerating activity; C3b, C3b binding site; C3d, C3d binding site; GAG, glycosaminoglycan binding site.

We have previously published functional studies on VUS in complement factor I (*CFI*) in patients with a TMA that facilitated establishing their clinicopathologic relevance (9, 10). We have also characterized complement factor H (*CFH*) and *CFI* variants identified in patients with age-related macular degeneration (AMD), a leading cause of blindness in the developed world. These findings helped explain the early onset of advanced AMD in the patients as well as the high burden of disease in their families (10, 11). We now present studies on the functional characterization of rare genetic variants and single nucleotide polymorphisms (SNPs) in *CFH* in our kidney transplant cohort (Figure 1). This study highlights how a systematic and comprehensive analysis can help to address the challenges described above, fill the knowledge gap relative to underlying mechanism of disease, and facilitate therapeutic decisions in a kidney transplant population. Our work also emphasizes several additional key concepts relative to limitations of the computational predictive models, the role of multiple *CFH* variants (common and rare), the impact of concomitant variants in *CFI* and membrane cofactor protein (*MCP, CD46*), and modifications due to underlying diseases including especially lupus.

## Materials and Methods

### Study Population

Patients diagnosed with a TMA or with biopsy-proven C3G between 2015 and 2020 at Washington University School of Medicine (WUSM) were evaluated. These patients were identified during the pretransplant evaluation at our center or were referred to us from other centers. Patients with a TMA included those with a biopsy-proven diagnosis in their native kidney or those who developed a *de novo* TMA after transplantation in the renal allograft. The diagnosis of C3G was based on the 2013 consensus report guidelines which required intensity of C3 staining at least two orders of magnitude greater than any other immunoreactant (i.e., IgG, IgM, IgA, and C1q) on a scale of 0–3 (including 0, trace, 1+, 2+, 3+) in native kidney or allograft (11). This research followed the tenets of the Declaration of Helsinki. The study was approved by the institutional review board at Washington University School of Medicine in St. Louis, MO. Informed consent was obtained from each subject.

### Sequencing

Next generation sequencing was performed by the Genomic and Pathology Services (GPS) at WUSM (12). The clinically validated aHUS/C3G next generation sequencing-based assay is derived from the Agilent Clinical Research Exome capture reagent (Agilent Technologies, Inc., Santa Clara, CA), with analysis of all exonic sequences for 13 genes (*ADAMTS13, C3, CD46, CFB, CFH, CFHR1, CFHR2, CFHR3, CFHR4, CFHR5, CFI, DGKE, THBD, MMACHC*, and *PLG*). CFHR3-CFHR1 copy number was assessed using multiplex ligation-dependent probe amplification (MLPA). All variants are reported according to Human Genome Variation Society (HGVS) nomenclature and classified based on the guidelines established by the joint consensus of the ACMG and the Association of Molecular Pathology (AMP). Based on these standards, variants are classified as: Level 1: pathogenic; Level 2: likely pathogenic; Level 3: variants of uncertain clinical significance (VUS); Level 4: likely benign; and Level 5: benign. The *CFH* reference wild-type (WT) sequence NM_000186.4 was employed for variant calling.

### Molecular Engineering of Factor H CCP 1–8 and CCP 15–20 Fragments

Preparation of the FH CCP 18–20 vector has been described (14). A two-step DNA synthesis method was used to construct the FH CCP 1–8 and CCP 15–20 vectors (15). The CCP 1–8 and CCP 15–20 fragments were generated by PCR with a C-terminal 6× His tag. These products were used as templates for a second PCR reaction in which the outer primer contained an N-terminal signal peptide and an EcoRI restriction site. The PCR products were subsequently digested with EcoRI and BamHI and then ligated with the pSG5 vector. The fidelity of FH CCP 1–8, CCP 15–20, and CCP 18–20 pSG5 vectors was verified by DNA sequencing. Primers used for PCR amplification are listed in Supplementary Table 1.

### Preparation and Expression of FH Variants

The FH variants (Figures 1B–D) were produced using the QuikChange XL site-directed mutagenesis kit (Agilent Technologies, Santa Clara, CA). Each *CFH* cDNA clone was sequenced. The variants were transfected into human embryonic kidney 293T cells and DMEM was replaced with serum-free OptiMEM® (Invitrogen, NY). Transient transfections were performed using the Xfect reagent (Takara Bio USA, Mountain View, CA). Supernatants were collected after 72 h, concentrated 80×, and stored in aliquots at −80°C (9).

### Quantifying FH Variants by ELISA and Western Blotting

**Figure 2:**
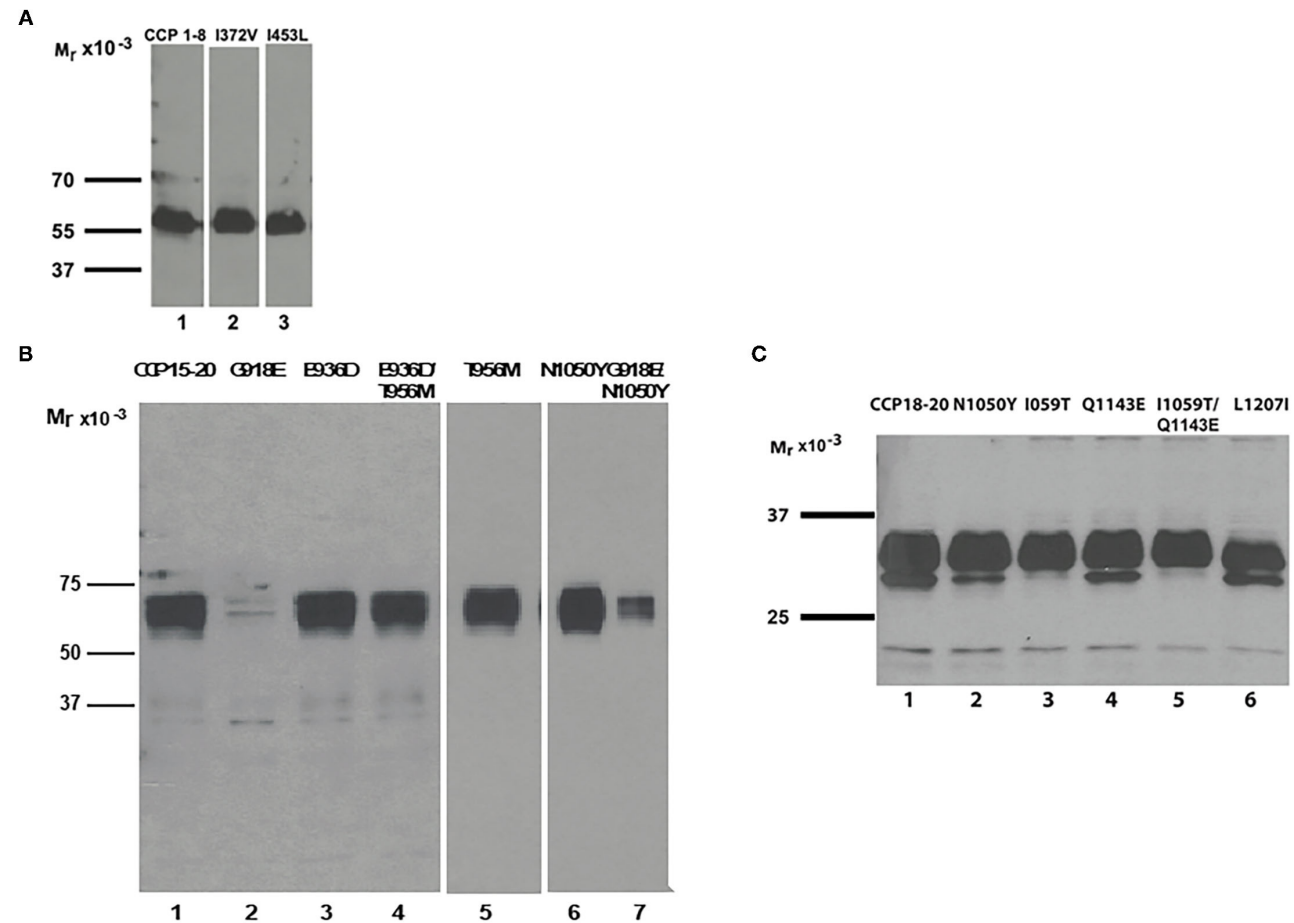
Recombinant expression of CFH variants. Western blots of supernatants from transfected wild-type and variant constructs. **(A)** Recombinant expression of variants I372V (lane 2) and I453L (lane 3) shows secretion comparable to wild type CCP 1–8 (lane 1). **(B)** Wild type CCP 15–20 as control (lane 1). Secretion of variant protein G918E is undetectable (lane 2). The SNP N1050Y displays higher secretion than WT (lane 6). The combined variant G918E/N1050Y has markedly reduced secretion (lane 7). Variant T956M has normal secretion (lane 5). Combined variant T956M/E936D and the SNP E936D also demonstrate normal secretion (lanes 3 and 4). **(C)** Wild type CCP 18–20 as control (lane 1). The SNPs N1050Y, I1059T, Q1143E, and variant L1207I show normal secretion (lanes 2–6).

The quantity of each recombinant FH variant protein was determined by ELISA (9, 16). The microtiter plates were coated with capture antibodies (Abs) (A254 for CCP 1–8; A229 for CCP 15–20, and CCP 18–20, Quidel) at 2 μg/ml overnight at 4°C and blocked for 1 h at 37°C (in 10 mM Tris, 150 mM NaCl pH 7.4, 1% BSA, and 0.05% Tween 20). The WT, variant fragments and purified human FH (Complement Technologies, TX) were diluted, incubated for 1 h at 37°C, and then washed with PBS pH 7.4 containing 0.05% Tween 20 (PBS-T). A polyclonal goat antiserum to human FH (A312, San Diego, CA) was employed to detect bound FH using a standard ELISA protocol (9, 16). For Western blots, supernatants were electrophoresed under reducing conditions using 4–20% SDS-PAGE, transferred to nitrocellulose, and then probed with 1:5,000 mouse Monoclonal TetraHis Ab (Qiagen, USA), followed by a 1:10,000 HRP goat anti-mouse IgG (Abcam, Cambridge, MA) (Figure 2).

### Functional Studies

The variants were produced as recombinant protein fragments of FH and their function was tested against the corresponding WT FH fragments and also the WT full-length protein. Patient serum was also used for fluid-phase and cell-surface assays. A genetic variant was considered to be deleterious if the protein was (a) not synthesized and/or not secreted *in vitro* (as measured by Western blot and ELISA) or (b) secreted in normal amounts but was dysfunctional based on any one of the assays outlined below.

#### Binding Assays

Variant and WT FH binding to C3b and heparin was assessed by ELISAs. Microtiter plates were coated with human C3b (37.5 nM for CCP 1–8; 15 nM for CCP 15–20 and CCP 18–20) or unfractionated heparin (2 mg/ml; Sigma-Aldrich) overnight at 4°C in NaHCO_3_ buffer [50 mM for C3b binding (17); 100 mM for heparin binding, pH 9.6) (18)], followed by blocking with 4% BSA in PBS-T (15, 19). Samples were diluted in ELISA buffer and incubated for 1 h at 37°C. Bound FH was detected as described previously (9, 16). On at least three occasions, binding assays were performed employing serially diluted samples.

#### Cofactor Activity

Factor H proteins were diluted in physiologic salt buffer (10 mM Tris pH 7.4, 150 mM NaCl). C3b (15 ng) was incubated at 37°C with purified FH (200 ng), equivalent moles of WT CCP 1–8 or variant FH protein, and FI (20 ng) in a total reaction volume of 20 μl. Kinetic analyses were performed at 0, 5, 10, and 20 min. The samples were electrophoresed on a 4–20% gradient Tris-glycine gel and then transferred to nitrocellulose for Western blotting (9).

#### Serum-Based Assays

These included the sheep red blood cell hemolytic assay, the C3b deposition assay and the C3G biomarker panel. These tests were conducted at the Molecular Otolaryngology and Renal Research Laboratories (MORL), Iowa (https://morl.lab.uiowa.edu/). The hemolytic assay measures complement-mediated lysis of sheep erythrocytes secondary to activation of the alternative pathway. The C3b deposition measures complement-mediated C3b deposition on the surface of cultured MES-13 cells as visualized using Alexa-488 labeled anti-C3 antibody.

#### Statistical Analysis

Statistical analyses were performed using Prism software version 8 (GraphPad). Comparisons between two groups were assessed using paired *t-*test (non-parametric). Comparisons among groups were performed using a one-way ANOVA with Dunnett's multiple comparison test (*P* < 0.05 was considered as significant).

## Results

A cohort of 76 kidney transplant patients (comprising 60 patients with a TMA and 16 with C3G) were identified and underwent genetic sequencing. Thirty-three of the 76 patients (~ 44%) carried a variant in a complement protein (Supplementary Table 2). Twenty-one of the 33 variants (~64%) were classified as a VUS. We have previously reported functional studies on VUS in *CFI* in this cohort that helped to establish their clinical significance (9). Variants in *CFH* were the focus of the current investigation. Ten rare genetic variants and four SNPs (E936D, N1050Y, I1059T, Q1143E) in *CFH* were identified (Supplementary Tables 2, 3). Of the 10 *CFH* variants, four were classified as pathogenic (C611Stop, C853R, c.619 + 1G>A, c.3493 + 1G>A), five as VUS (I372V, I453L, G918E, T956M, and L1207I) and one as likely benign (T1017I). We investigated the effects of the five VUS in *CFH* in eight patients. We also functionally characterized the SNPs (alone and in combination with a rare variant). Two patients in one family also carried concomitant variants in *CFI* (K441R, classified as likely benign) and *MCP* (c.287-2A>G, classified as pathogenic).

We hereby present “real-life” cases of eight patients with a detailed clinical history for each. Comprehensive antigenic, functional, and structural analyses were conducted to determine the underlying etiology of disease which was key for clinical decision-making regarding treatment and risk of recurrence.

### Complement-Mediated TMA Vs. Secondary TMA After Kidney Transplantation

#### Clinical History

##### Patient 1

A 45-year-old Asian female developed end-stage renal disease (ESRD) secondary to biopsy-proven lupus nephritis. She underwent a deceased donor kidney transplant (DDKT) in 2008, which was complicated on post-operative day 5 by a TMA in the allograft leading to a transplant nephrectomy (Supplementary Figure 1). The etiology of TMA was presumed to be the calcineurin inhibitor (CNI). Therefore, during the second DDKT in 2012, belatacept was used instead of a CNI. Two years later, the patient developed AKI requiring hemodialysis (HD). Allograft biopsy (not shown) revealed acute cellular rejection and endothelialitis which did not respond to conventional treatment with steroids and she remained dialysis dependent. Given the loss of two allografts due to TMA and pathologic evidence of endothelial cell injury, genetic testing for complement variants was performed during the evaluation for a third transplant (Table 1).

**Table 1:** Genetic, immunologic, and clinical data from the TMA/C3G cohort at Washington University School of Medicine (WUSM).

**Patients**	**Variants**	**MAF (%)**		**Allele count (gnomAD)/Number of homozygotes**	**C3 level (mg/dl)**	**C4 level (mg/dl)**	**Factor H (mg/dl)**	**FH autoantibody (≤22 unit/ml)**	**C3Nef (0.0–0.26)**	**Transplant history**
		**Total**	**Population**							
1	I372V	0.0008	0.011 (East Asian)	2/0	72 (83–185)	19.2 (12–54)	66.2 (37–68)	Negative	Negative	2 failed
2	I453L^*^	0.0032	0.012 (African American)	1/0	57 (90–180)	11 (10–40)	38.8 (37–68)	Negative	Negative	1 failed
3 - 5	G918E[Table-fn TN2]	0.0008	0.003 (Latino)	2/0	72.2 (79–152)	NT	47.1 (16–41.2)	Negative	Negative	None
6	T956M	0.13	0.17 (Caucasian)	366/2	68 (90–180)	NT	56.8 (37–68)	38	1.02	1 failed
7	T956M	0.13	0.17 (Caucasian)	366/2	105 (75–175)	25 (14–40)	79.9 (37–68)	Negative	0.3	Allograft functioning well
8	L1207I	Novel	NT	Not reported/NA	NT	NT	NT	Negative	NT	Donor candidacy declined

*Minor allele frequency (MAF) values were based on total and specific population frequency from Genome aggregation database (gnomAD).*

* *Laboratory tests showed characteristics of a TMA—anemia [hemoglobin, 7.7 g/dl (normal range, 12.0–15.0 g/dl); elevated LDH, 292 U/L (normal range, 100–250 U/L); low haptoglobin, 17 mg/dl (normal range, 30–200 mg/dl)]; thrombocytopenia [platelet count, 66 k/μl (normal range, 150–400 k/μl)], and AKI (creatinine, 8.94 mg/dl with baseline 1.5 mg/dl); schistocytes on peripheral smear (3–7/HPF).*

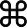
*Laboratory studies were consistent with a TMA—anemia [hemoglobin, 3.9 g/dl (normal range, 12–15.5 g/dl); elevated LDH, 1,723 U/L (normal range, 140–280 U/L); undetectable haptoglobin, <30 mg/dl (normal range, 50–220 mg/dl)]; thrombocytopenia [platelet count, 71 k/μl (normal range, 150–450 k/μl)], and AKI (creatinine, 7.0 mg/dl with baseline 0.7 mg/dl); and schistocytes on peripheral smear (3–4/HPF). NT, not tested. I, isoleucine; V, valine; L, leucine; G, glycine; E, glutamic acid; T, threonine; M, methionine*.

##### Patient 2

A 36-year-old African American female developed ESRD secondary to biopsy-proven lupus nephritis. She received a DDKT which failed within 1 year and she returned to dialysis. Allograft biopsy demonstrated arterioles with marked mucoid intimal edema and fragmented red cells. Laboratory results suggested a TMA (Table 1). Therefore, she underwent genetic testing during the evaluation for a second transplant.

For both patients described above, there was no family history of kidney disease. ADAMTS13 activity was normal and elevated levels of anti-phospholipid antibodies (APL Abs) or FH autoantibodies were not detected. Testing for Shiga toxin was also negative.

#### Genetic Analysis

We identified rare heterozygous *CFH* variants, I372V in patient 1 and I453L in patient 2 (Table 1). I372V has been reported in gnomAD with a MAF of 0.0008% in East Asians only and I453L with a MAF of 0.0032% in an individual of African ancestry. They were classified as VUS due to their rarity and lack of functional data or other evidence to support pathogenicity.

#### Structural Analysis

**Figure 3:**
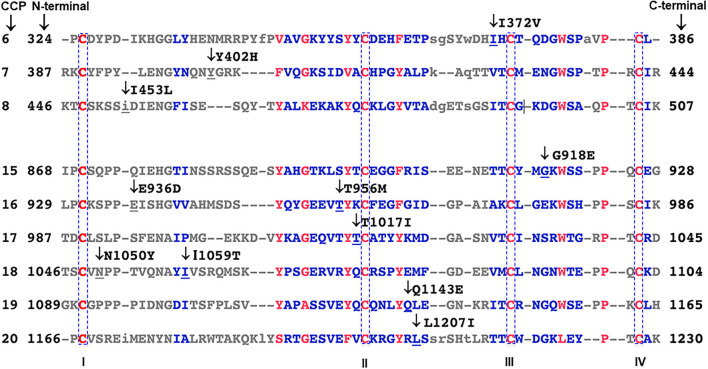
Amino acid sequence alignment of complement control protein (CCP) repeats in FH. Sequence alignments are generated with NCBI protein BLAST software based on position-specific scoring matrix (PSSM). Red to blue color scale represents the degree of conservation; red represents highly conserved residues; blue represents less conserved residues. Upper-case residues are aligned, lower-case residues are unaligned. Mutated residues of FH in the cohort are indicated with arrows. Two disulfide bonds form in each CCP, one between cysteine I and III and the other between cysteine II and IV (red and highlighted).

**Figure 4:**
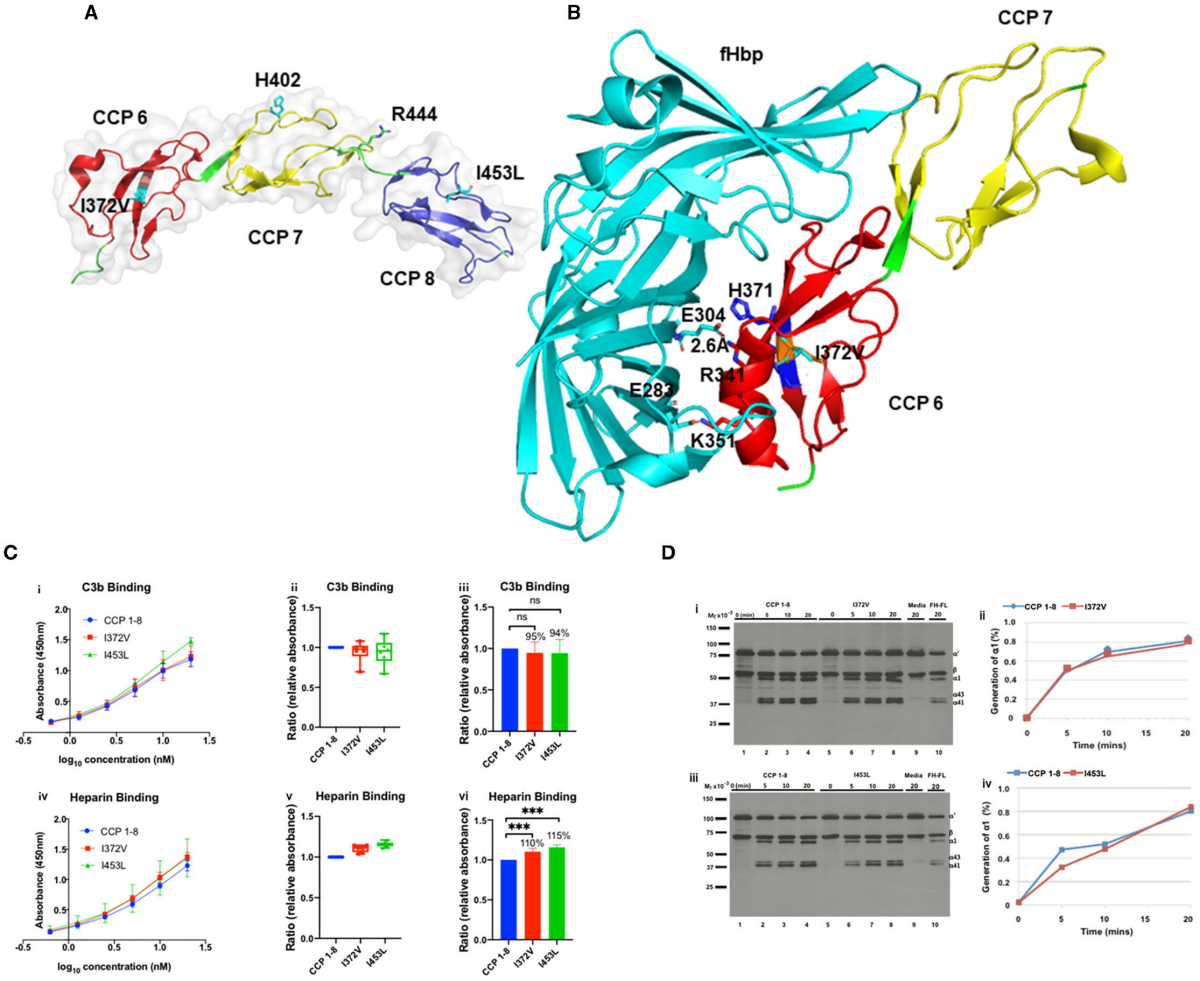
Structural analysis of I372V and I453L **(A)** The overall structure of FH CCP 6–8 is shown in a cartoon representation (PDBID:2UWN) (20). CCP 6 is in red, CCP 7 in yellow, and CCP 8 in blue. H402 and the two rare variants, I372V and I453L, are in cyan. Crystallographic structural data indicates that CCP 6–8 contain four glycosaminoglycan (GAG) binding sites (20). The primary binding site is H402 (20); the other three putative binding sites are H360 (not shown) in CCP 6, R341 in CCP 6 (in **B**), and R444 in CCP 7 (colored green at the linker between CCP 7 and CCP 8). **(B)** Interface structure of FH CCP 6–7 in complex with FH binding protein (fHbp) derived from *Neisseria meningitidis* (PDBID: 2W80) (21). Side chains from both proteins involved in forming salt bridges across the interaction surface are shown. The putative heparin binding site is composed of H337 (not shown), R341 and H371 in CCP 6 (21). E304 and R341 form a salt bridge over a distance of approximately 2.6Å. E304 and E283 in cyan from fHbP; R341 and K351 from CCP 6 in red; the variant I372V in orange is flanked by H371 and H373 in blue. Structures are prepared using PyMol. Functional analysis of I372V and I453L **(C)** (i, iv) Absorbance is plotted against logarithmic protein concentrations. (ii, v) Box-and-whisker plots demonstrating C3b and heparin binding (by ELISA) of I372V and I453L compared to WT. Relative absorbance (RA) is computed as absorbance of the variant divided by absorbance of the WT. Bottom of the box shows the 25^th^ percentile, the line within the box indicates the median, and the top of the box shows the 75^th^ percentile. (iii, vi) Representation of C3b and heparin binding of I372V and I453L compared to WT using bar graphs. For C3b binding the *P*-value for the percentage differences of I372V and I453L compared to WT were 0.69 and 0.65, respectively. For heparin binding, the *P*-value for the percentage differences of I372V and I453L compared to WT were both <0.001. Data represent three separate experiments with bars corresponding to SEM (standard error of mean). ns, no significant difference; ***P < 0.001. **(D)** (i, iii) Fluid-phase C3b cofactor activity (CA) of I372V and I453L assessed by the cleavage of purified C3b to iC3b compared to WT. The percentage of alpha chain remaining and the generation of α1 indicates the cleavage of C3b to iC3b. A kinetic analysis of CA was conducted at 0, 5, 10, and 20 min. Cleavage rate is measured by densitometric analysis of the generation of α1 relative to the β chain. Representative Western blot is shown. (ii, iv) Densitometric quantification of the Western blot. Lane 9 represents a negative control employing concentrated supernatant in the presence of FI but absence of FH. Lane 10 is a positive control using full-length FH (FH-FL). Upon comparison to WT FH, there is no difference in CA of I372V and I453L.

The I372 residue is a moderately conserved amino acid in CCP 6 (Figure 3) that is spatially adjacent to a putative GAG binding site in CCP 6–8 of the FH crystal structure (PDBID:2UWN and 2W80) (Figures 4A,B) (20, 21). The I372V substitution involves two hydrophobic amino acids. Both crystal structures (PDBID 2UWN and 2W80) demonstrate that the hydrophobic side chain of I372 is buried inside the CCP 6 protein core, away from the interacting surface. However, the alteration to a smaller hydrophobic residue V may influence the external surface of CCP 6 and its binding interactions through an internal structural rearrangement.

Sequence alignments of mammalian CCP 8 revealed that I453 is moderately conserved in 10 mammalian sequences (Figure 3) (22). Although I453 is spatially away from R444 (a putative heparin binding site) (19) (Figure 4A), the substitution of I to L may influence the external surface of CCP 8 and its binding interactions.

#### Antigenic and Functional Analyses

Factor H antigenic levels in both patients were in the normal range (Table 1). Secretion of the recombinantly produced variants (I372V and I453L) and their binding affinities to C3b were comparable to those of WT and full-length FH (Figures 2, 4C; Table 2). Cofactor activity in our standard assay with the recombinant proteins was also normal (Figure 4D). However, the variants showed a modest increase in binding affinity for heparin compared to WT. Given the aggressive nature of the TMA, loss of multiple allografts, rarity of the variants, and the structural data supporting a possible deleterious effect, we conducted further investigation using serum-based assays. The samples were sent to MORL, Iowa for the C3b deposition and sheep red blood cell hemolytic assays. The C3b deposition assay was abnormal for I372V (40–60% C3b deposition detected; reference range, negative, MORL, Iowa) and the sheep hemolytic assay was abnormal for I453L (20–40% hemolysis detected; normal <3%, MORL, Iowa).

**Table 2:** Summary of the functional analyses for *CFH* variants.

**Patient (s)**	**Variant**	**Location**	**Recombinant secretion (μg/ml)**	**C3b binding**	**Heparin binding**	**Cofactor activity**	**Cell-surface regulation using patient serum**	**ACMG interpretation**	**Modified interpretation (based on functional and structural analysis)**
1	I372V	CCP 6	Comparable to WT	N	N	N	Defective (C3b deposition assay)	VUS	Deleterious
2	I453L	CCP 8	Comparable to WT	N	N	N	Defective (sheep red cell hemolytic assay)	VUS	Deleterious
3, 4	G918E	CCP 15	Decreased	ND	ND	__	Not done	VUS	Deleterious
3, 4	G918E/N1050Y	CCP 15/18	Decreased	ND	ND	__	Not done	VUS	Deleterious
6	T956M/E936D	CCP 16	Comparable to WT	N	N	__	Not done	VUS	Normal function
7	T956M	CCP 16	Comparable to WT	MD	N	__	Not done	VUS	Likely benign
8	L1207I	CCP20	Comparable to WT	N	N	__	Not done	VUS	Normal function

*Secretion of I372V (5.9 μg/ml) and I453L (6.2 μg/ml) was comparable to WT CCP1–8 (4.0 μg/ml) (P > 0.05, SEM 1.15) as measured by ELISA. C3b binding and cofactor activity were normal but cell surface regulation was defective for both I372V and I453L. Variant G918E was not secreted. Secretion of SNP N1050Y (4.1 μg/ml) was comparable to WT CCP 15–20 (3.6 μg/ml) (P < 0.05, SEM 0.1). Secretion of G918E/N1050Y was markedly reduced compared to WT. Secretion of T956M (2.6 μg/ml) and T956M/E936D (3.5 μg/ml) were comparable with WT CCP 15–20 (3.6 μg/ml) (P = 0.7855, SEM 0.1). Secretion of L1207I was comparable to WT CCP 18–20 (2 μg/ml) (P > 0.05, SEM 0.16). ACMG, American College of Medical Genetics; VUS, variant of uncertain significance. Patient 5 did not undergo genetic testing due to financial/insurance issues. N, normal; ND, not done; MD, marginally decreased. I, isoleucine; V, valine; L, leucine; G, glycine; E, glutamic acid; T, threonine; M, methionine*.

### Implications

Patients 1 and 2 represent examples of post-transplant TMA in patients with SLE in which a rare complement genetic variant was identified. This clinical scenario is challenging for clinicians to determine if such patients have a primary complement-mediated TMA or a secondary SLE-associated or a transplant-related TMA or a combination of the above. Our analyses demonstrate that the variants I372V and I453L are produced normally and thus have no quantitative defect but are deleterious due to a qualitative defect based on the distinctly abnormal serum-based assays. The structural analysis also supports a likely deleterious effect.

However, given that the recombinant FH protein demonstrated normal results but serum-based assay revealed impaired function in the C3b deposition or the sheep red blood cell hemolytic assays led us to consider other possibilities. The question was raised that elevated FHR levels in the serum might be the cause of impaired FH function of complement regulation. To address this issue, we re-examined the next generation sequencing data which included CFHR1, CFHR2, CFHR3, CFHR4, and CFHR5 genes. Additionally, CFHR3-CFHR1 copy number was assessed. Neither patient carried a variant in the CFHR genes. A heterozygous CFHR3-1 deletion was identified in patient carrying the variant I453L but not in patient carrying the variant I372V. Since altered FHR levels have not been associated with a heterozygous CFHR3-1 deletion, we think that it is less likely that the patients have elevated FHR levels but this possibility cannot entirely be ruled out.

To summarize, these two patients did not develop TMA in the native kidney but developed an early and aggressive disease in the allograft in the presence of a trigger(s) that included transplant surgery and/or rejection. Overall, these data help to clarify that the TMA in these two patients is likely complement-mediated. Therefore, in both cases it was recommended that eculizumab be administered at the time of the next kidney transplant.

### Familial Postpartum TMA Syndrome With Multiple Genetic Variants

#### Clinical History

##### Patient 3 (Sister 1, Proband)

In 2015, a 25-year-old Hispanic female presented 10 days after an uncomplicated vaginal delivery with laboratory results consistent with a TMA (Table 1). Treatment with prednisone and plasma exchange (PE) was unsuccessful and HD was initiated. ADAMTS13 activity and APL Abs were normal. Eculizumab was started after kidney biopsy confirmed a TMA (not shown). Hemodialysis was discontinued after 8 weeks. Two months later, she developed flash pulmonary edema due to acute mitral valve regurgitation secondary to an anterior leaflet perforation, which necessitated a mitral valve replacement. The patient is currently doing well with stable kidney function. In retrospect, the proband's two sisters had similar clinical presentations.

##### Patient 4 (Sister 2)

In 2014, this 24-year-old presented 7 days postpartum after an uncomplicated vaginal delivery with anemia, thrombocytopenia, and AKI. She was unsuccessfully treated with PE and HD had to be initiated. The proband diagnosis was established 1 year later, leading to aHUS being considered in sister 2 and eculizumab was initiated. Her creatinine (Cr) initially stabilized at 2–2.5 mg/dl and was 0.9 mg/dl in December 2020. However, she did not continue eculizumab due to health insurance issues. Since discontinuation of eculizumab, the renal function has remained stable, but she has developed hematuria and proteinuria (1.2 g).

##### Patient 5 (Sister 3)

In 2006, this 18-year-old presented 7 days postpartum after an uncomplicated vaginal delivery with anemia, thrombocytopenia, and AKI. Similar to her sisters, she did not respond to PE and HD was initiated. The course was complicated by heart failure due to severe mitral and tricuspid regurgitation. She was not treated with eculizumab as aHUS was not considered until after the diagnosis had been made in her sisters (8 years later). She currently remains on dialysis.

Family history in this kindred is significant for a paternal grandmother who lost 10 of 12 pregnancies due to miscarriages, and a paternal aunt who is currently on dialysis (Supplementary Figure 2). No medical history is available for the father. The mother delivered five healthy children (the three sisters mentioned above and two brothers) with no postpartum complications.

#### Genetic Analysis

The proband was a compound heterozygote for two non-synonymous variants in *CFH*, G918E, and N1050Y (Table 1; Supplementary Table 3). The G918E variant is rare being identified in two individuals in gnomAD, one each of European and Latino ancestry, and has also been reported in a patient with a TMA (23). The G918 residue is highly conserved across species and *in silico* predictions point to a deleterious effect of the G918E variant. Due primarily to a lack of *in vitro* studies, it was classified as a VUS.

The N1050Y SNP occurs at a position that is not highly conserved and *in silico* analyses yield inconsistent predictions. This variant has been associated with reduced AMD risk (24) and is not enriched in aHUS (25, 26). Based on these criteria, the N1050Y variant was classified as benign.

A heterozygous splice-site variant (c.287-2A>G) was identified in the *MCP* (C*D46*) (27). *In vitro* studies are consistent with an exon skipping effect predicted to result in protein truncation (27, 28). This variant has been identified in aHUS, and is classified as a pathogenic variant based on functional, computational and population evidence (2, 29, 30).

A heterozygous missense variant, K441R, in *CFI* was also identified and previously described as likely benign (31). Sister 2 carries the same genetic variants in *CFH* and *MCP* as the proband but does not carry the variant in *CFI*. Unfortunately sister 3 did not undergo genetic testing.

#### Structural Analysis

**Figure 5:**
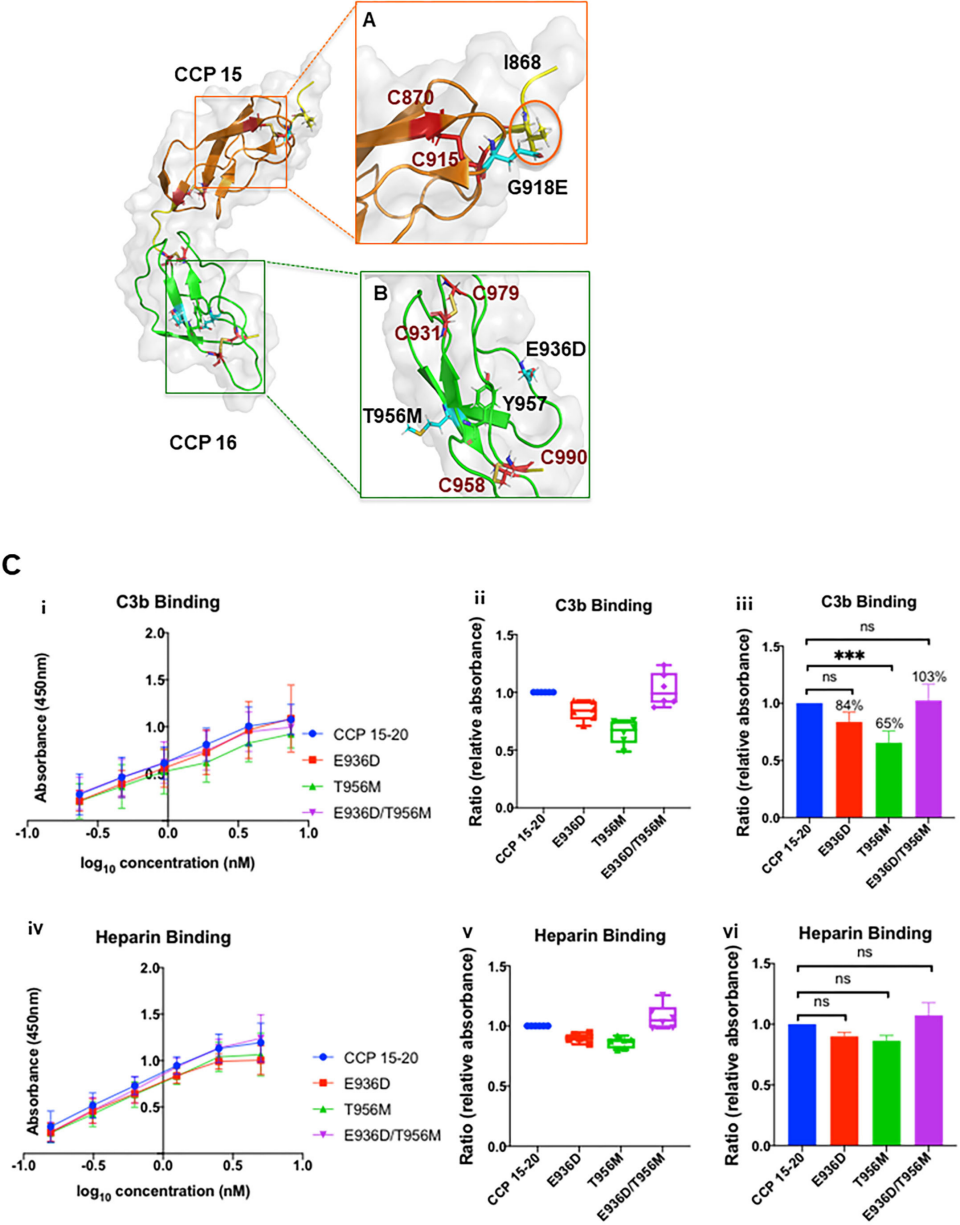
Structural analysis of G918E, E936D, and T956M. Nuclear magnetic resonance spectroscopy solution structure of CCP 15–16 (PDB ID: 1HFH) (32). **(A)** The highest frequency rotamer of the G918E side chain and its clashing residue I868 is highlighted in the orange circle. **(B)** The highest frequency rotamer of the T956M and E936D side chains is highlighted in cyan. C958 and C990 form a disulfide bond next to the Y957 residue to stabilize the CCP 16 tertiary structure. E936D and T956M are located on the protein surface, spatially distant from the disulfide bond. CCP 15 (orange); CCP 16 (green); linker between CCP 15 and 16 (yellow); cysteine side chains involved in disulfide bond formation (red); G918E, E936D, and T956M variants (cyan sticks, zoomed and reoriented in inset box). Functional analysis of T956M and E936D. **(C)** (i, iv) Absorbance is plotted against logarithmic protein concentrations. (ii, v) Box-and-whisker plots demonstrating C3b and heparin binding (by ELISA) of E936D, T956M, and combined variant E936D/T956M compared to WT. (iii, vi) Representation of C3b and heparin binding of E936D, T956M, and E936D/T956M compared to WT using bar graphs. Compared to WT there was no difference in heparin binding for E936D, T956M, or E936D/T956M. T956M showed ~35% decrease in C3b binding compared to WT (*P* < 0.001). Data represent three separate experiments with bars corresponding to SEM. ***P < 0.001.

G918 is located in CCP 15 between the highly conserved C915 and W920 residues (Figure 3). Substitution of G with E introduces a negatively charged side chain at the main surface loop between β-strands 6 and 7 (Figure 5A). This alteration could generate side chain clashes between E918 and I868 that perturb the conformation of the adjacent C870–C915 disulfide bond and thereby disrupt FH protein folding.

N1050 is located in CCP 18 (Figure 3), close to the interdomain linker between CCP 17 and CCP 18, which could alter the inter-CCP domain arrangements. Because Y has a larger relative size than N, its presence in the variant may modulate the overall folded-back CCP domain structure but should not affect FH stability.

#### Antigenic and Functional Analyses

Factor H and FI antigenic levels in the proband were normal, but MCP expression was low (Table 1). Secretion of the recombinantly produced variant protein G918E was undetectable (Figure 2B; Table 2). By contrast, the recombinant N1050Y SNP displayed higher secretion than WT (Table 2). We produced the combined variant G918E/N1050Y and showed that the secretion was markedly reduced (Figure 2B). We also recombinantly produced the CFI variant K441R which displayed normal secretion and no defect in cofactor activity (not shown).

#### Implications

These cases represent pregnancy-associated TMA in a family that carries genetic variants in multiple complement proteins (FH, MCP, and FI). The MCP mutation is pathogenic given the low expression in our patient and having been reported previously as having low expression on granulocytes (27). Our structural and functional data establish that the *CFH* variant G918E is also deleterious because it abolishes FH protein expression. The proband though had a normal FH level. We speculate that in our patient this may be due to enhanced protein production by the N1050Y SNP, likely consistent with N1050Y location on the other allele. This hypothesis is supported by the fact that another patient carrying G918E had a reduced FH level of 168 mg/l (normal range 180–420 mg/l; personal communication, Richard Smith, Iowa).

These results indicate that TMA etiology in these patients is complement-mediated based on defective *CFH* and *MCP* variants. The risk of recurrent TMA after a kidney transplant in patients carrying an MCP mutation is low since the allograft comes with a normal MCP. However, since the two sisters also harbor the dysfunctional *CFH* mutation, they are at higher risk for recurrent disease.

We propose that complement dysregulation may have caused multiple miscarriages in the paternal grandmother and ESRD in the paternal aunt. The presentation of acute heart failure (AHF) due to valvular disease in two sisters is unusual and has not been reported previously. We propose that the mechanism underlying AHF is TMA-associated vasculitis with thrombus formation leading to valvular dysfunction.

Our work highlights a unique postpartum aHUS presentation with a remarkable phenotype involving the renal and cardiac systems and illustrates the putative role of multiple variants in disease etiology (33).

### C3 Glomerulopathy Associated With and Without a Monoclonal Gammopathy

#### Clinical History

##### Patient 6

A 67-year-old Caucasian female with biopsy-proven C3GN in the setting of a monoclonal gammopathy (elevated IgG kappa on serum protein electrophoresis and immunofixation) underwent a DDKT in 2016. She had an elevated FH autoantibody and a C3 nephritic factor (C3Nef; 1.02, normal range 0.0–0.26). Prior to transplant, she was treated with bortezemib, cyclophosphamide, and dexamethasone, which reduced the M-protein (≥90%) and led to negative tests for both autoantibodies. She developed recurrent C3GN in 2018 (Supplementary Figure 3). A bone marrow biopsy revealed involvement by the previously diagnosed plasma cell neoplasm. A year later the patient died secondary to refractory congestive heart failure.

##### Patient 7

A 28-year-old Caucasian male with biopsy-proven C3GN underwent a living unrelated kidney transplant from his wife in Feb 2020. He had an elevated C3Nef (0.3, normal range 0–0.26, Blood Center of Wisconsin) associated with a high Bb level (2.7 mg/l, normal <1.2 mg/l) and an elevated soluble C5b-9 (1.51 mg/l, normal <0.3 mg/l). The alternative pathway complement functional assay (AH50) was low (72%, normal 75–170) as well as the properdin level (7.5 mg/l, normal 10–33 mg/l). A monoclonal gammopathy was not detected (Table 1). There was no family history of kidney disease. At last follow-up in March 2021, Cr was stable at 1.2 mg/dl with no proteinuria.

#### Genetic Analysis

A rare heterozygous variant T956M was identified in patients 6 and 7 (Table 1). This variant is located at a position that is not highly conserved but has been reported previously in patients with aHUS (34–36). Most *in silico* tools predict that this variant is tolerated; however, MutationAssessor and Polphen2-Hdiv predict a damaging effect. It was classified as a VUS due to a lack of functional studies. Patient 6 also carried a SNP, E936D, which is common across multiple populations and is associated with a predisposition to aHUS (Supplementary Table 3) (37). *In silico* tools however predict that E936D is benign.

#### Structural Analysis

The T956 residue in CCP 16 is less conserved and adjacent to the highly conserved Y957 residue (Figure 3). The hydrophobic Y957 side chain is packed in the CCP 16 interior, whereas the hydrophilic T956 side chain is exposed on the protein surface (Figure 5B). The substitution of hydrophilic T with the long hydrophobic side chain M in the T956M variant may perturb the beta sheet orientation, and therefore alter CCP 16 folding.

The E936 residue in CCP 16 is less conserved in multiple CCP sequence alignments (Figure 3). E and D are both negatively charged hydrophilic amino acids in the E936D variant, and the substitution is highly tolerated without interfering with protein folding (Figure 5B).

#### Antigenic and Functional Analysis

Factor H antigenic levels in both patients were normal (Table 1). Secretion of the recombinantly produced T956M variant was comparable to that of WT (Figure 2; Table 2). The variant had slightly reduced affinity for C3b binding compared to WT, but unchanged affinity for heparin binding (Figure 5C). Defective C3b binding was corrected with the double mutant E936D/T956M.

#### Implications

These two patients with C3GN carry a genetic variant (T956M) and acquired defects in the form of autoantibodies (C3Nef and/or FH). Our analyses demonstrate that T956M may have a modest functional defect due to ~30% decrease in C3b binding. However, the function improved when combined as variant E936D/T956M (Figure 5C). The T956M variant was also previously reported in an aHUS patient and functional data showed no defect (36). Thus, while the structural analysis proposes a possible deleterious effect, functional analysis by us and others establish that T956M is likely benign. Therefore, the development of autoantibodies (FH and C3Nef) in patient 6 is most likely related to the underlying monoclonal gammopathy, a well-known association of C3G (38). Patient 7 persistently harbors an elevated C3Nef and an abnormal complement biomarker profile (due to an unclear etiology) despite being on immunosuppression and thus is at risk for recurrent C3GN.

### Living Donor Kidney Transplantation in aHUS

#### Clinical History

##### Patient 8

A 63-year-old Caucasian male was evaluated as a kidney donor for his 36-year-old son. The son had developed ESRD at age 33 and was given a diagnosis of possible aHUS. He was initially treated with plasmapheresis and subsequently switched to eculizumab. The son underwent genetic testing which identified a heterozygous CFHR3-1 deletion. The father underwent genetic testing as part of donor evaluation work-up. This case was referred to us from an outside hospital to assist with genetic interpretation of the VUS identified in the father as described below.

#### Genetic Analysis

A novel heterozygous missense variant L1207I was identified. This variant is located within a poorly conserved nucleotide position in CCP 20 (Figure 3) (39). *In silico* functional prediction indicated a tolerated effect of this alteration. This variant though was categorized as a VUS due to the paucity of functional studies and lack of other pathogenicity supporting evidence. Two SNPs (I1059T and Q1143E) and a homozygous CFHR3-1 deletion were also identified. The father or son did not carry FH-autoantibodies.

#### Structural Analysis

**Figure 6:**
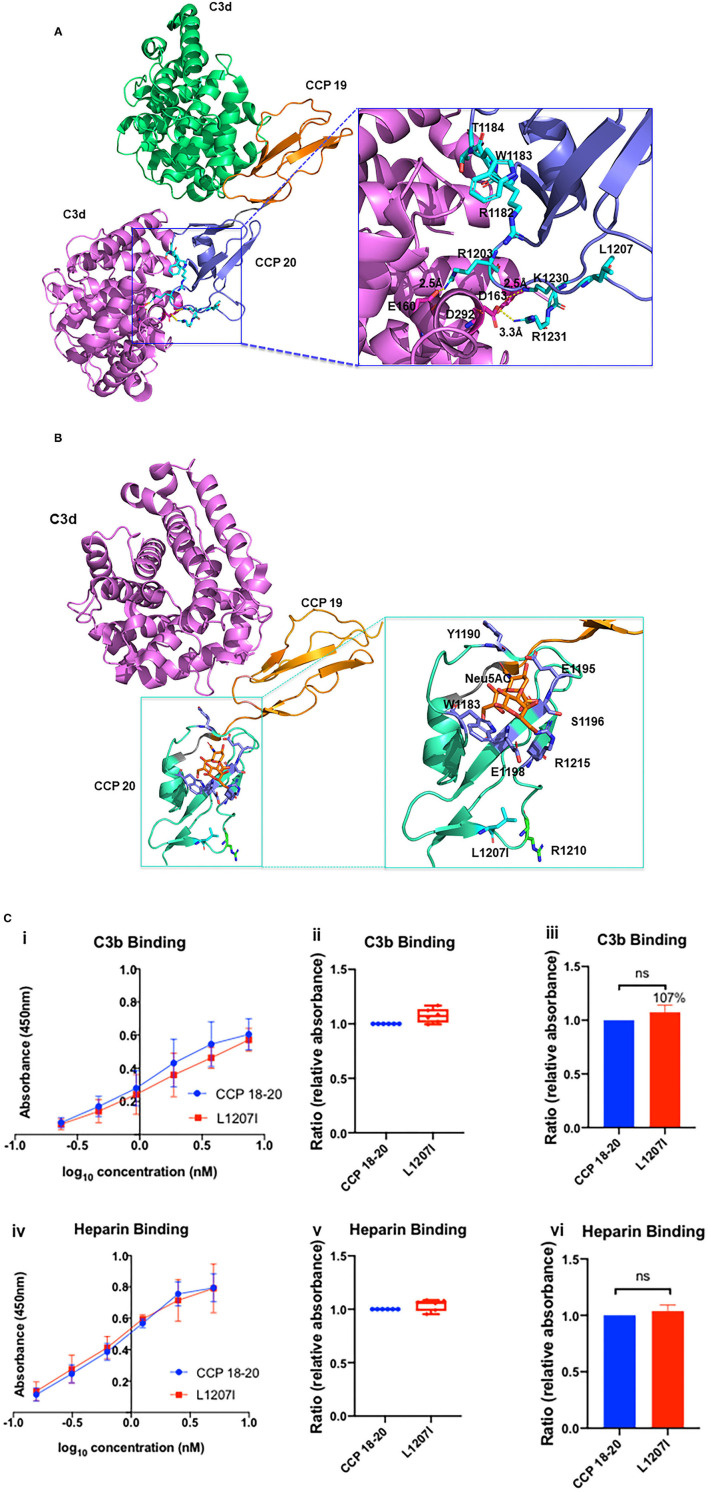
Structural analysis of L1207I. **(A)** Structure of the CCP 19–20:C3d complex containing one molecule of CCP 19–20 and two molecules of C3d (PDB ID 2XQW, green and purple) (40). Residues located at the CCP 20 interface are annotated and shown as cyan sticks. Residues located at the C3d interface are shown as magenta sticks. Close-up view of the CCP 20-C3d interface is shown and reoriented in the inset box. R1182, W1183, and T1184 mutations shown as cyan sticks were reported in aHUS cases (40). Functional study of W1183L demonstrated impaired interaction with surface-bound C3b (14). E160 and R1203, D163 and K1230, D292 and R1231 form salt bridges with distances of approximately 2.5, 2.5, and 3.3 Å, respectively. L1207 (in cyan) is located away from the C3d-CCP 20 interaction surface. **(B)** Sialic acid binding site on complement CCP 20 (PDB ID 4ONT) (41)—C3d (magenta); CCP 19 (orange); CCP 20 (green); α-N-acetylneuraminic acid (Neu5AC, orange stick). L1207 (cyan) is spatially distant from the binding surface between Neu5AC and CCP 20. R1210 mutations (green sticks) have been reported in aHUS. (42). Functional analysis of L1207I **(C)** (i, iv) Absorbance is plotted against logarithmic protein concentrations. (ii, v) Box-and-whisker plots demonstrating C3b and heparin binding (by ELISA) of L1207I compared to WT. (iii, vi) Representation of C3b and heparin binding of L1207I compared to WT using bar graphs. There is no difference in C3b or heparin binding for L1207I compared to WT. Data represent three separate experiments, bars correspond to SEM.

CCP 20 interacts with the C3d cleavage fragment of C3b and GAGs (14). C3d binds electrostatically with CCP 20. The interacting surface consists of a positively charged pole formed by R1203, K1230, and R1231 from CCP 20, and a negatively charged cleft formed by E160, D163, and D292 from the C3d moiety of C3b (Figure 6A) (40). CCP 20 has a GAG binding site that is formed by a hydrophobic pocket. The key amino acids identified in this region are L1181, R1182, W1183, S1191, E1195, S1196, E1198, and R1215 (Figure 6B) (14). L1207 does not appear to directly coordinate either C3d or GAG binding. L and I are two hydrophobic and highly interchangeable amino acids, and the substitution of L to I likely does not alter the overall conformation of the C3b and heparin binding sites.

#### Antigenic and Functional Analysis

Secretion of the recombinantly produced variant protein in the supernatant was normal compared to that of WT (WT, 1.1 μg/ml; L1207I, 1.07 μg/ml) (Figure 2C). Functional studies confirmed that the variant had no significant effect on either C3b or heparin binding compared to that of WT (*P* > 0.05) (Figure 6C). Functional analysis of the SNPs (I1059T and Q1143E) was also performed and demonstrated no defect (Supplementary Figure 4).

#### Implications

This case is challenging because it involves a living donor transplant for a father-son pair. The son (potential recipient) has a presumptive diagnosis of aHUS with no identified genetic variants, whereas the healthy father (potential donor) has a rare genetic variant (L1207I) and SNPs in *CFH*. Our analyses showed that L1207I is not functionally or structurally deleterious and probably explains why the father did not develop kidney disease. In this case, donor candidacy was declined since functional analysis of the VUS was not available at the time of transplantation. More importantly, a definitive diagnosis of aHUS and its etiology in the son remains unknown.

Living-related donor kidney transplant is generally contraindicated for patients with aHUS (43) because a nephrectomy may trigger TMA in the genetically susceptible donor. Although donor genetic analysis may be performed, some recipients have more than one mutation and genetic testing does not reveal a variant in the complement gene in approximately one-third of patients with aHUS (such as in this case). We may consider living-related donor transplants in patients who carry a known pathogenic mutation in a complement protein that causes disease in the patient if the donor tests negative for that mutation. The recipient and living donor should participate in the decision-making process after they understand the risks and benefits.

## Discussion

We describe clinical cases of eight patients with a TMA or C3G from our kidney transplant cohort. In each case, we were faced with the challenge of determining the disease etiology and recurrence risk because of the presence of confounding risk factors in their clinical history. Moreover, these patients carried genetic variants in *CFH* that were classified as a VUS. Since there was a lack of information in the literature about the functional impact of these variants, we employed an integrative approach by utilizing the clinical, genetic, antigenic and structural data in addition to the functional analyses, to further define the implications for each variant (5).

Our work highlights a multi-modality approach that substantially facilitated: (i) Establishing the diagnosis of a complement-mediated TMA in a complex clinical setting (lupus) with multiple triggers (Patients 1 and 2) (ii) Defining the role of multiple deleterious variants in a unique postpartum TMA presentation featuring a three-sister kindred with multiple organ system involvement (Patients 3, 4, and 5). (iii) Illustrating the prominent role of acquired factors (monoclonal gammopathy, C3 nephritic factor, and FH autoantibody) in C3G even though a rare variant was identified but demonstrated normal function (Patients 6 and 7) (iv) Formulating an informed therapeutic decision about a potential living donor transplantation in the setting of a TMA (Patient 8).

Our results point out potential problems in using the ACMG guidelines to interpret variants. These guidelines are designed for Mendelian diseases and heavily weigh population frequency. Consequently, despite functional data demonstrating no defect, the interpretation for many of the genetic variants described in our cases remained a VUS primarily due to the rarity of the variant. Based on these results, we suggest that modifications to the ACMG framework for diseases with variable penetrance, such as aHUS, be considered. For combined variants, we speculated but could not confirm if they were present on the same or different alleles. One way to further address this issue is to test family members to determine whether variants are shared by other affected and unaffected relatives. In our cases, the family samples were not available for sequencing.

There are two quite informative studies on *CFH* variants in aHUS and C3G by Merinero et al. that are particularly worth noting here. In the first one, the authors report functional characterization of *CFH* variants using FH purified from plasma samples of FH Y402H heterozygous carriers (36). This was an elegant approach to obtaining purified full-length FH which was available for 7 of 12 variants. While our manuscript was being prepared for submission, a second comprehensive study reported a functional assessment for 105 *CFH* variants associated with aHUS (44). In this study, full length FH was prepared recombinantly by ThermoFisher Scientific (Regensburg, Germany). Of note, four of the five VUS we describe here have not been reported previously including in this recent publication. The one overlapping variant, T956M, demonstrated similar results to our data. Also, the SNPs (E936D, N1050Y, I1059T, Q1143E) have been reported and results of our functional data are comparable (Supplementary Figure 4) (44). Additionally, we demonstrate that the SNP E936D may have a potentially protective role since it helped improve the C3b binding for rare variant, T956M. We used FH fragments which have been shown by us (16) and others (45–47) to be highly predictive of the results obtained with full-length protein (36, 44). Nevertheless, the availability of commercially available full-length recombinant FH and the serum-based assays is an advance that should be taken advantage of.

**Figure 7:**
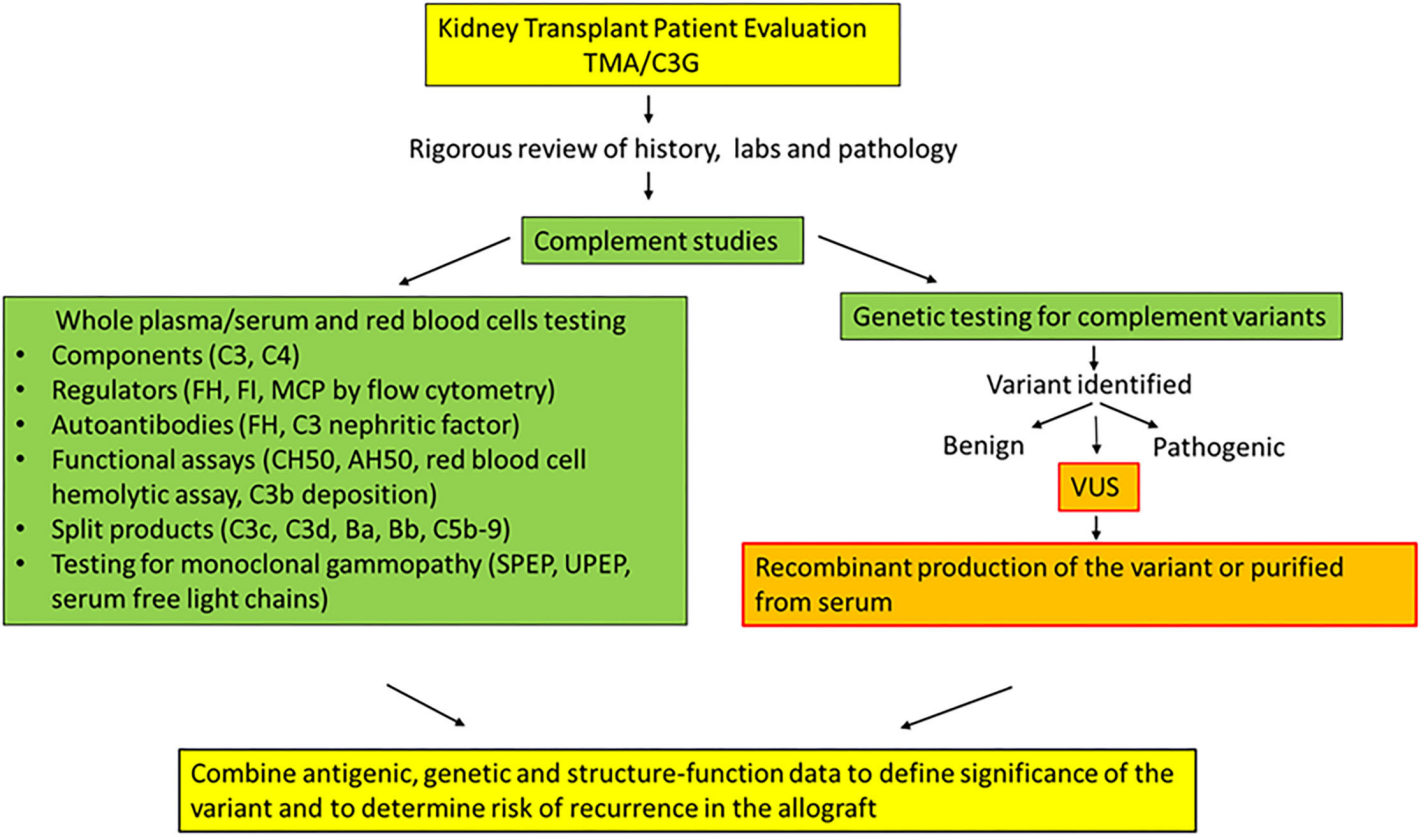
Algorithm with recommended testing and steps to define clinical significance of genetic variants in patients with TMA or C3G.

In summary, there are challenges in reconciling genetic data with clinical management. The strategy of recombinant protein production followed by systematic antigenic, functional, and structural assessment remains the gold standard (Figure 7). We predict that such functional assessments will become more widely available in the future and will be critical to address knowledge gaps and identify the etiology and pathogenic mechanisms in patients with complement-mediated kidney diseases, especially in a complex clinical setting of kidney transplantation. The ongoing development of complement biomarkers and novel assays will provide clinicians with the tools to stratify patients for targeted therapy and adapt precision medicine for genetic kidney diseases.

## Data Availability Statement

The original contributions presented in the study are publicly available. These data can be found here: ncbi.nlm.nih.gov/clinvar/, SCV001905506–SCV001905512.

## Ethics Statement

The studies involving human participants were reviewed and approved by Institutional Review Board Washington University School of Medicine in St. Louis, MO. The patients/participants provided their written informed consent to participate in this study. Written informed consent was obtained from the individual(s) for the publication of any potentially identifiable images or data included in this article.

## Author Contributions

ZR, AJ, and JA conceived and designed the experiments, analyzed the data, and interpreted results. ZR performed the experiments. ZR and SP conducted the structural analysis. LL-G classified variants in accordance with ACMG 2015 criteria. ZR prepared the figures and drafted the manuscript. ZR, SP, LL-G, JA, and AJ edited the manuscript. All authors contributed to the article and approved the submitted version.

## Funding

This study was supported by the Barnes-Jewish Hospital Foundation Fund (AJ). Research reported in this publication was also supported by NIGMS, NEI, and NIAMS of the National Institutes of Health under award numbers R35 GM136352, R01 EY028602, and R21 AR076534 (JA). The manuscript was edited by the Scientific Editing Service of the Institute of Clinical and Translational Sciences at Washington University, which is supported by an NIH Clinical and Translational Science Award (UL1 TR002345).

## Conflict of Interest

AJ reports serving on the scientific advisory boards of Alexion Pharmaceuticals and Novartis Pharmaceuticals and being a consultant for Gemini Therapeutics and Chinook Therapeutics. AJ is a principal investigator on a trial by Apellis pharmaceuticals. JA reports serving as a consultant for Celldex Therapeutics, Clinical Pharmacy Services, Kypha Inc., Achillion Pharmaceuticals Inc., and BioMarin Pharmaceutical Inc. and having stock or equity options in Compliment Corporation, Kypha Inc., Gemini Therapeutics, and Q32 BIO INC. The remaining authors declare that the research was conducted in the absence of any commercial or financial relationships that could be construed as a potential conflict of interest.

## Publisher's Note

All claims expressed in this article are solely those of the authors and do not necessarily represent those of their affiliated organizations, or those of the publisher, the editors and the reviewers. Any product that may be evaluated in this article, or claim that may be made by its manufacturer, is not guaranteed or endorsed by the publisher.
