# Supplementary material for: Clinicopathologic Implications of Complement Genetic Variants in Kidney Transplantation
Source: Front Med (Lausanne). 2021 Nov 29;8:775280. doi: 10.3389/fmed.2021.775280 (PMC8666976; doi:10.3389/fmed.2021.775280)
Supplement: Supplementary file 1 [file Data_Sheet_1.PDF]

Supplementary Material

Supplementary Figures

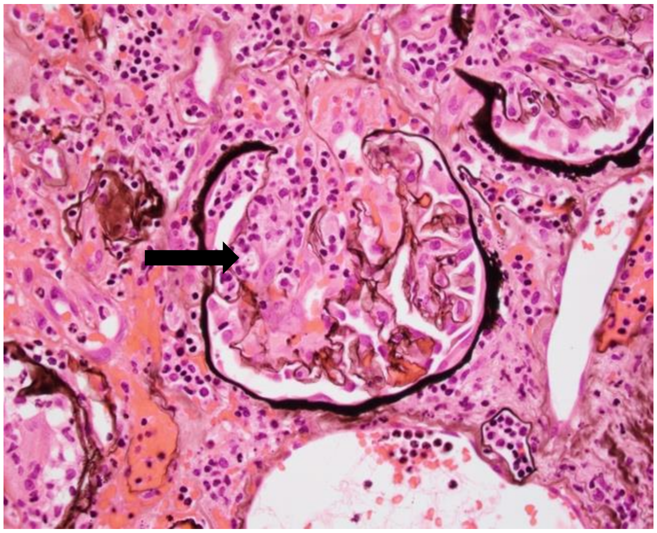

Figure S1. The glomeruli showed diffuse global mesangiolysis (thick arrow). Fibrin thrombi were observed in several glomeruli. Focal cortical necrosis was present. Small arteries and arterioles showed severe endothelialitis with focal fibrinoid necrosis of the arterial wall.

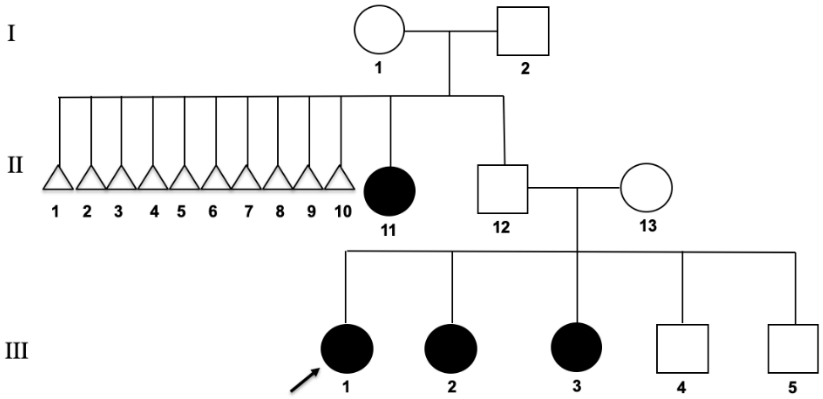

Figure S2. Pedigree of the family for the three-sister kindred (Case 3,4,5). Arrow, proband; squares, male; circles, female. Black symbols indicate persons with kidney disease; white symbols represent unaffected persons; triangular symbols represent miscarriages.

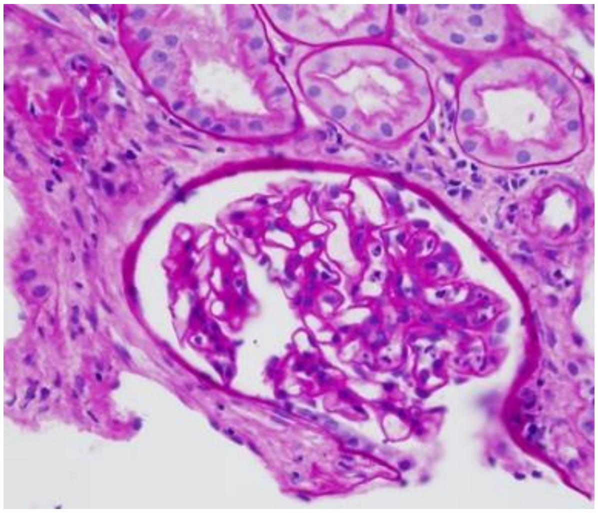

A. Light micrograph showing segmental neutrophilic glomerulitis.

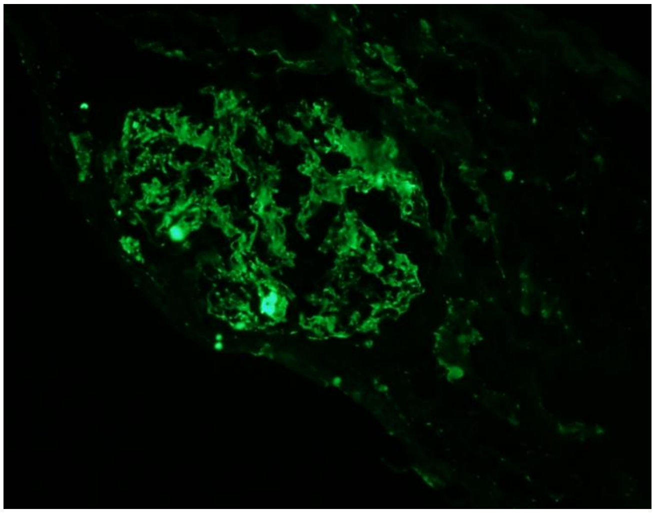

B. The glomeruli demonstrating predominant mesangial granular staining positive for C3 (3+ on a scale of 0-3) by immunofluorescence microscopy.

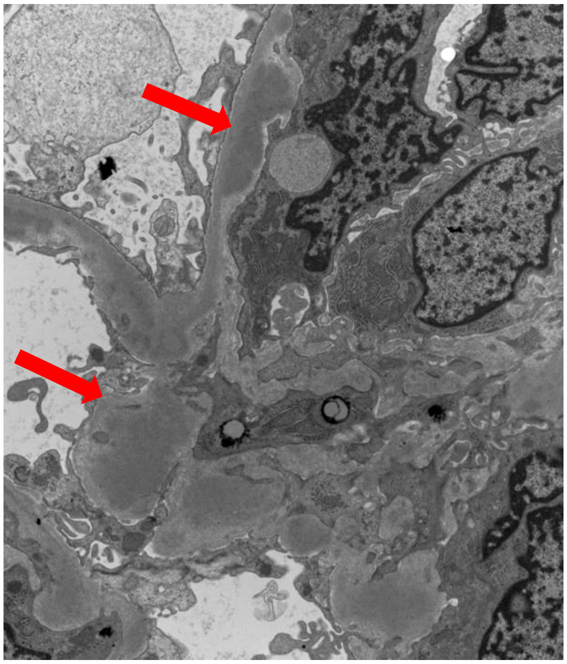

C. Electron dense deposits (red arrows) identified by electron microscopy. The deposits have no organized substructure and were seen in mesangial, intramembranous, occasional subepithelial and subendothelial locations.

Figure S3. Renal histopathological findings in C3G (patient 6)

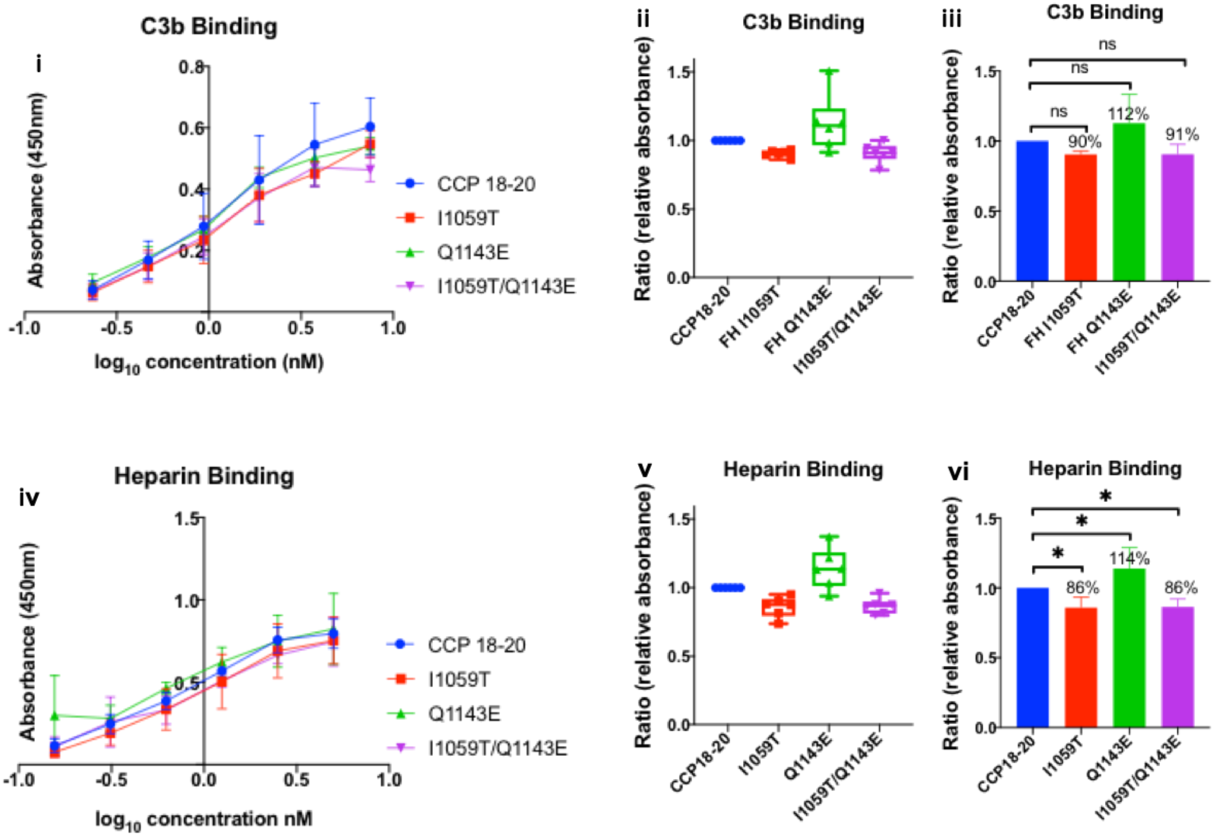

Figure S4. Binding analysis of I1059T, Q1143E, and I1059T/Q1143E variants. (i, iv) Absorbance is plotted against logarithmic protein concentrations of WT CCP18–20, I1059T, Q1143E, and I1059T/Q1143E. (ii, v) Box-and-whisker plots demonstrating C3b and heparin binding (by ELISA) of I1059T, Q1143E and I1059T/Q1143E compared to WT. (iii, vi) Representation of C3b and heparin binding compared to WT using bar graphs. Values for the differences in the percentages of I1059T and I1059T/Q1143E were $P < 0.02$ and $P < 0.03$, respectively. Data represent three separate experiments, bars correspond to SEM. ns, not significant.

Supplementary Tables

Table S1. Primers used for PCR amplification of CCP 1–8 and CCP 15–20 constructs.

Region	PCR primers (5′ → 3′)
CCP 1–8	Outer forward primer:<br>5′-CCGGAATTCATGAGACTTCTAGCAAAGATTATTTGCCTTATGTTATGGGCTATTTGTGTA-3′<br>Inner forward primer:<br>5′-GGGCTATTTGTGTAGCAGAAGATTGCAATGAA-3′<br>Reverse primer:<br>5′-GCGGATCCTTAGTGGTGGTGGTGGTGGTGAGATTTAATGCA-3′
CCP 15–20	Outer forward primer:<br>5′-CCGGAATTCATGAGACTTCTAGCAAAGATTATTTGCCTTATGTTATGGGCTATTTGTGTA-3′<br>Inner forward primer:<br>5′-GGGCTATTTGTGTAGCAGAAAAAATTCCATGTTCACAACCACCTCAGAT-3′<br>Reverse primer:<br>5′-CGGGATCCCTAGTGGTGGTGGTGGTGGTGTCTTTTTGCACAAGTTGGAT-3′

Table S2. Genetic complement abnormalities reported in 76 patients from the TMA/C3G cohort at Washington University School of Medicine. *CFH*, complement factor H; *CFI*, complement factor I; *MCP*, membrane cofactor protein or CD46; *CFB*, complement factor B. *Functional studies for VUS in CFI have been published (1).

Genetic abnormality	Number of pathogenic variants	Number of VUS	Number of likely benign
**CFH**	**4**	**5**	**1**
**CFI**	**2**	**8***	**–**
**C3**	**2**	**6**	**–**
**MCP**	**3**	**1**	**–**
**CFB**	**–**	**1**	**–**

Table S3. Single nucleotide polymorphisms (SNPs) in the TMA/C3G cohort at Washington University School of Medicine. Serum FH levels were within normal range for these individuals. n, the number of patients in our cohort carrying the SNPs

Mutation	MAF (%)					Prediction
	Total	African	East Asian	European	Latino	
**E936D (n=14)**	**19.9**	**5.8**	**50.5**	**16.8**	**35.4**	**Benign**
**N1050Y (n=8)**	**1.5**	**2.7**	**0.005**	**2.0**	**0.8**	**Benign**
**I1059T (n=2)**	**0.7**	**6.9**	**0.0**	**0.03**	**0.4**	**Benign**
**Q1143E (n=3)**	**1.0**	**10.3**	**0.0**	**0.02**	**0.5**	**Benign**

Supplementary Reference

1. Java A, Pozzi N, Love-Gregory LD, Heusel JW, Sung YJ, Hu Z, et al. A Multimodality Approach to Assessing Factor I Genetic Variants in Atypical Hemolytic Uremic Syndrome. *Kidney Int Rep*. 4 (2019) 1007-17. doi:10.1016/j.ekir.2019.04.003
